# Enlarged splenic volume predicts poor survival and is associated with inflammatory imbalance in patients with diffuse large B-cell lymphoma

**DOI:** 10.1007/s44313-026-00138-1

**Published:** 2026-05-08

**Authors:** Zongjian Qiu, Rifeng Jiang, Shunquan Wu, Rong Zhan, Xiaomei Hu, Shaoyuan Wang

**Affiliations:** 1 Fujian Provincial Key Laboratory On Hematology, Fujian Institute of Hematology, Fujian Medical University Union Hospital, Fuzhou, Fujian Province People’s Republic of China; 2 Department of Hematology, Fujian Medical University Union Hospital, Xinquan Road No.29, Fuzhou, Fujian Province 30001 People’s Republic of China; 3 Department of Radiology, Fujian Medical University Union Hospital, Fuzhou, Fujian Province People’s Republic of China; 4 Department of Pathology, Fujian Medical University Union Hospital, Xinquan Road No.29, Fuzhou, Fujian Province 30001 People’s Republic of China

**Keywords:** Prognostic factor, Inflammatory markers, Imbalanced anti-cancer immunology

## Abstract

**Purpose:**

The spleen plays a significant role in innate and adaptive immunity. We aimed to evaluate the prognostic significance of the splenic volume in patients with diffuse large B-cell lymphoma (DLBCL), research on which is limited.

**Methods:**

We retrospectively analyzed 175 patients diagnosed with DLBCL. Based on optimal thresholds, patients were stratified into small (< 185 cm^3^), medium (185–315 cm^3^), and large (≥ 315 cm^3^) splenic volume groups. Survival analysis was performed using Kaplan–Meier and Cox proportional hazards model.

**Results:**

A significant decrease in progression-free survival (PFS) (*P* < 0.0001) and overall survival (OS) (*P* < 0.0001) was observed in the large group that received cyclophosphamide, hydroxydaunorubicin, vincristine, and prednisone (CHOP) without or with rituximab (R-CHOP) treatment. Among the 139 patients who received R-CHOP, significant adverse effects on PFS (*P* < 0.0036) and OS (*P* < 0.0035) were observed in the large group. Univariate and multivariate analyses demonstrated that enlarged splenic volume (≥ 315 cm^3^) was associated with decreased PFS (*P* < 0.0001, *P* < 0.01, respectively) and OS (*P* < 0.001, *P* < 0.01, respectively). In terms of peripheral blood inflammatory markers, patients with enlarged splenic volume (≥ 315 cm^3^) exhibited lymphocytopenia, increased monocyte-to-lymphocyte ratio (MLR), increased neutrophil-to-lymphocyte ratio (NLR), and higher red blood cell distribution width (RDW) compared to that in those with smaller splenic volumes (*P* < 0.001, *P* < 0.001, *P* = 0.01, and *P* < 0.005, respectively).

**Conclusion:**

Splenic volume exceeding 315 cm^3^ is a poor prognostic factor for PFS and OS in patients with DLBCL. An enlarged splenic volume may be associated with compromised anti-cancer immune responses and aberrant inflammatory conditions.

**Supplementary Information:**

The online version contains supplementary material available at 10.1007/s44313-026-00138-1.

## Introduction

Diffuse large B-cell lymphoma (DLBCL) is the predominant form of non-Hodgkin lymphoma, accounting for approximately 30–40% of newly diagnosed B cell tumors globally [1]. Although the prognosis of the disease has been improved by rituximab-based immunochemotherapy, the current standard regimen of cyclophosphamide, hydroxydaunorubicin, vincristine, prednisone, and rituximab (R-CHOP) yields a cure rate of only 50–70% in these patients, with 15–20% exhibiting resistance to all forms of chemotherapy and 30–40% experiencing relapse or disease progression [2]. Multiple factors are currently used to predict the prognosis of DLBCL.

The largest lymphoid organ, the spleen, contains various immune cells, including both innate and adaptive immune cells [3]. Extramedullary hematopoiesis (EMH) in the spleen, which serves as a supplementary hematopoietic site supporting myelopoiesis, is triggered during periods of bodily stress or in response to immune challenges, such as infection and tumor induction, as demonstrated in a mouse model [3–6]. Spleen-derived immune cells promote tumor growth, immune evasion, and metastasis, often at the expense of tumor-reactive lymphoid cells [7, 8]. In our clinical practice, we have encountered a subset of patients diagnosed with DLBCL who exhibited splenomegaly and lacked fluorodeoxyglucose (FDG) uptake during initial evaluation via Positron Emission Tomography-Computed Tomography (PET-CT) scanning. Investigations on the correlation between splenic volume and outcomes in patients with DLBCL are limited. Consequently, this retrospective study explored the association between splenic volume and outcomes in patients with DLBCL.

## Materials and methods

### Study population

A total of 175 patients with DLBCL, who received treatment at the Fujian Medical University Union Hospital between January 2015 and June 2017, were included in this study. All patients with newly diagnosed de novo DLBCL, aged ≥ 14 years, and with available baseline imaging (PET CT or contrast enhanced CT) were considered for inclusion. No minimum number of treatment cycles was required. Patients were excluded if they (1) had HIV-associated DLBCL, transformed DLBCL, DLBCL with liver cirrhosis, collagen diseases, autoimmune hemolytic anemia, other hematological disorders, or a history of malignancy; (2) were lost to follow-up without any documented outcome after treatment initiation; and (3) received fewer than four cycles of treatment, provided that they did not experience early death or disease progression during this period (e.g., due to patient preference, socioeconomic barriers, transfer for care, or non-fatal toxicity). Subsequent follow-up was conducted at Fujian Medical University Union Hospital from January 2015 to September 2023.

### Clinical data collection

The clinical characteristics and demographic data of the patients, including sex, age, Ann Arbor classification of disease stage, International Prognostic Index (IPI), lactate dehydrogenase (LDH) level, complete blood cell count, Han’s classification of pathology, Eastern Cooperative Oncology Group (ECOG) score, and number of involved extranodal sites, were obtained from medical records. The initial evaluation and treatment response assessment criteria were based on the Lugano classification system. Progression-free survival (PFS) was defined as the duration from diagnosis to disease progression, relapse, death from any cause, or the last follow-up date. Overall survival (OS) was defined as the duration from diagnosis to death from any cause or the last follow-up date. Follow-up data were collected at 3-month intervals within the first year, at 6-month intervals during the second year, and annually thereafter.

### Splenic volume calculation

The splenic volume was determined during the initial radiological assessment by outlining the spleen surface on all PET-CT or contrast-enhanced CT slices. Subsequently, the cumulative planimetric area was calculated and multiplied by the slice thickness to obtain splenic volume.

### Criteria for assessing spleen involvement and bone marrow in patients with DLBCL

The criteria for assessing bone marrow and spleen involvement were based on the Lugano classification [9]. PET-CT imaging was performed in 123 (70.3%) patients to determine disease stage. Positive PET-CT findings for spleen involvement included diffuse uptake, a solitary mass, miliary lesions, or nodular lesions. Additionally, 52 (29.7%) patients underwent contrast-enhanced CT at baseline before treatment initiation, with a solitary mass, nodules, or a vertical length of > 13 cm indicating splenic involvement. Bone marrow aspiration and biopsy were conducted for all patients diagnosed with DLBCL. Bone marrow involvement was identified by FDG-avid skeletal lesions or confirmed histologically after bone marrow aspiration and biopsy.

### Statistical analysis

In this study, the cutoff point for splenic volume was determined based on OS using X-tile software [10]. The categorical variables were presented as frequencies in percentages. Categorical variables were analyzed using the chi-squared or Fisher's exact tests. Continuous variables were analyzed using the Mann–Whitney U or Wilcoxon rank-sum test. The correlation between splenic volume and anthropometric values (height and weight) was assessed using the Spearman’s correlation coefficient. The Kaplan–Meier method was used to estimate the survival curves for PFS and OS, and the log-rank test was used to compare them. The hazard ratio (HR) and corresponding 95% confidence interval (CI) for the risk effect of splenic volume were estimated using a stratified Cox regression model, with splenic volume as the sole explanatory variable. Univariate and multivariate Cox regression analyses were used to determine risk factors for OS and PFS. Two-tailed *p*-values were reported, and a *P*-value less than 0.05 was considered statistically significant. Statistical analysis was conducted using IBM SPSS Statistics v. 21.0 (SPSS Inc., Chicago, IL, USA) and R software (version 4.3.0).

## Results

### Patients’ characteristics

X-title software was used to determine the threshold for splenic volume. Based on this threshold, splenic volume was categorized into three groups: small (< 185 cm3), medium (≥ 185 cm3 and < 315 cm3), and large (≥ 315 cm^3^). Table 1 shows the baseline clinical characteristics of the three groups. Among the 175 patients included in this study, 43 (24.6%) exhibited significant splenic enlargement. In the large group, 20 (46.5%) patients did not have splenic involvement as confirmed by PET-CT or contrast-enhanced CT (Fig S1). No statistically significant differences were observed in age, sex, weight, height, Han’s classification of pathology, and extranodal disease site (≥ 1) across the three groups of patients. Spearman’s rank correlation coefficient was used to analyze the associations between weight and splenic volume, as well as between height and splenic volume. Regression analysis revealed a weak, non-significant correlation between height and splenic volume (r = 0.051, *P* = 0.503), as well as between weight and splenic volume (r = 0.089, *P* = 0.240) (Fig S2). However, when comparing the large group to the medium and small groups respectively, a higher proportion of patients in the large group exhibited poor performance status (ECOG ≥ 2, 55.8% vs. 31.6% vs. 14.7%, *P* < 0.001), elevated LDH (LDH > upper limit of normal, 81.4% vs. 45.6% vs 32.0%, *P* < 0.001), advanced stage (Ann Arbor stage III/IV, 81.4% vs. 57.9% vs. 34.7%, *P* < 0.001), higher international prognostic index (IPI ≥ 3, 69.8% vs. 42.1% vs. 28.0%, *P* < 0.001), higher probability of bone marrow involvement (14.0% vs. 5.3% vs. 1.3%, *P* < 0.05), and a higher likelihood of spleen involvement (53.5% vs. 14.0% vs. 4.0%, *P* < 0.001).

**Table 1:** Baseline characteristics of patients in different groups

		Splenic volume size		
Clinical characteristics	Small (n = 75) (42.9%)	Medium (n = 57) (32.5%)	Large (n = 43) (24.6%)	*P*-value
Weight (Kg) Median (Min, Max)	57.0 (40.0, 84.0)	60.0 (42.0, 90.0)	60.0 (47.0, 88.0)	0.403
Height (cm) Median (Min, Max)	163 (145, 180)	165 (145,180)	170 (145, 180)	0.540
Age (years), n (%)				0.267
< 60	41 (54.7%)	39 (68.4%)	27 (62.8%)	
≥ 60	34 (45.3%)	18 (31.6%)	16 (37.2%)	
Sex, n (%)				0.060
Male	35 (46.7%)	38 (66.7%)	26 (60.5%)	
Female	40 (53.3%)	19 (33.3%)	17 (39.5%)	
Han’s classification, n (%)				0.426
GCB	34 (45.3%)	22 (38.6%)	21 (48.8%)	
non-GCB	38 (50.7%)	35 (61.4%)	21 (48.8%)	
unclassified	3 (4.0%)	0 (0%)	1 (2.3%)	
Ann Arbor stage, n (%)				< 0.001
I-II	49 (65.3%)	24 (42.1%)	8 (18.6%)	
III-IV	26 (34.7%)	33 (57.9%)	35 (81.4%)	
Radiology, n (%)				0.639
PET-CT	50 (66.7%)	41 (71.9%)	32 (74.4%)	
CT	25 (33.3%)	16 (28.1%)	11 (25.6%)	
Splenic involvement, n (%)				< 0.001
Yes	3 (4.0%)	8(14.0%)	23 (53.5%)	
No	72 (96.0%)	49(86.0%)	20 (46.5%)	
Bone marrow involvement, n (%)				0.013
Yes	1 (1.3%)	3 (5.3%)	6 (14.0%)	
No	74 (98.7%)	54 (94.7%)	37 (86.0%)	
Elevated LDH (> ULN), n (%)				< 0.001
Yes	24 (32.0%)	26 (45.6%)	35 (81.4%)	
No	51 (68.0%)	31 (54.4%)	8 (18.6%)	
Extranodal site number, n (%)				0.364
< 1	32 (42.7%)	30 (52.6%)	17 (39.5%)	
≥ 1	43 (57.3%)	27 (47.4%)	26 (60.5%)	
IPI, n (%)				< 0.001
0 ~ 2	54 (72.0%)	33 (57.9%)	13 (30.2%)	
3 ~ 5	21 (28.0%)	24 (42.1%)	30 (69.8%)	
ECOG, n (%)				< 0.001
< 2	64 (85.3%)	39 (68.4%)	19 (44.2%)	
≥ 2	11 (14.7%)	18 (31.6%)	24 (55.8%)	
Therapy, n (%)				0.176
Chemotherapy	12 (16.0%)	11 (19.3%)	13 (30.2%)	
Immuno-chemotherapy	63 (84.0%)	46 (80.7%)	30 (69.7%)	

*Abbreviations*: *GCB* germinal center B cell-like, *non-GCB* non-germinal center B cell-like, *PET-CT* positron emission tomography-computed tomography, *CT* computed tomography, *LDH* lactate dehydrogenase, *ULN* upper limit of normal, *IPI* international prognostic index, *ECOG* Eastern Cooperative Oncology Group

### Outcomes of patients in the cohort

**Fig. 1:**
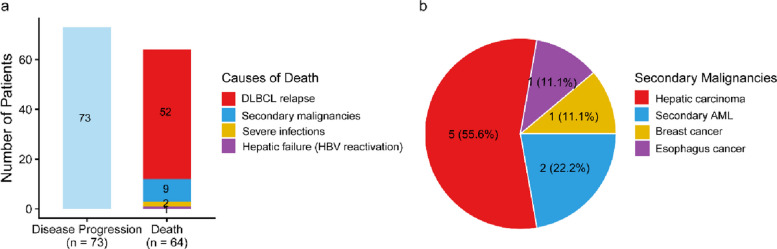
Patient outcomes and etiologies of mortality. **a** Incidence of disease progression and death in the study cohort (n = 175) and distribution of all causes of death (left), **b** A detailed composition of secondary malignancies, which occurred after DLBCL treatment. DLCBL, diffuse large B-cell lymphoma

**Fig. 2:**
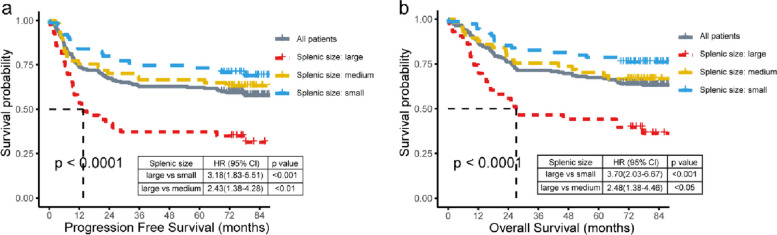
Kaplan–Meier curves of progression-free survival (PFS) (**a**) and overall survival (OS) (**b**) for 175 patients who received the CHOP regimen with or without rituximab in different splenic size groups: small (blue), medium (yellow), and large (red), and the pooled patients (gray). CHOP, cyclophosphamide, hydroxydaunorubicin, vincristine, and prednisone

**Fig. 3:**
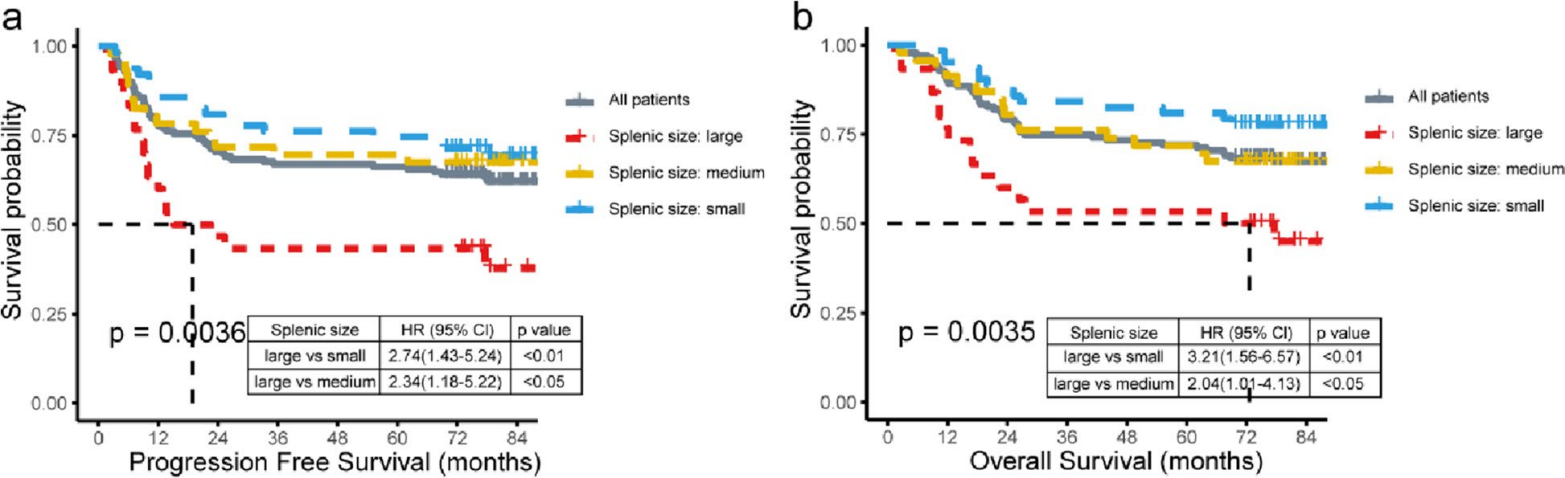
The Kaplan–Meier curves of progression-free survival (PFS) (**A**) and overall survival (OS) (**B**) for 139 patients treated with the R-CHOP regimen in different splenic size groups: small (blue), medium (yellow), and large (red), and the pooled patients (gray). R-CHOP, cyclophosphamide, hydroxydaunorubicin, vincristine, and prednisone with rituximab

In this study, the follow-up duration was 76.0 months (range: 0.1–104.1). Of the 175 patients in the cohort, 139 (79.4%) received the R-CHOP regimen, and 36 (20.6%) received the CHOP regimen. Among the 175 patients, 73 (41.7%) experienced disease progression and 64 (36.6%) died, including 52 patients dying from DLBCL relapse (of which three patients died from central nervous system relapse) and nine from secondary malignancies (hepatic carcinoma: five, secondary acute myeloid leukemia: two, breast cancer: one, and esophageal cancer: one) (Fig. 1). Additionally, two patients succumbed to severe infections (severe sepsis: one, pneumonia-related respiratory failure: one), and one patient died from hepatic failure caused by reactivation of the hepatitis B virus**.** Among the entire cohort of 175 patients, the 5-year PFS and OS rates were 62.3% (95% CI, 55.5–69.9) and 67.4% (95% CI, 60.8–74.7), respectively (Fig. 2). Specifically, among the 139 patients who received the R-CHOP regimen, the 5-year PFS and OS rates were 66.2% (95% CI, 58.8–74.5) and 71.9% (95% CI, 64.8–79.8), respectively (Fig. 3).

### Impact of different splenic sizes on survival of patients with DLBCL

In the entire cohort of 175 patients, Kaplan–Meier analysis revealed that the large group had significantly shorter median PFS (13.6 months; 95% CI, 9.4–77.4) and OS (27.2 months; 95% CI, 16.5–NA) compared to those in the small- and medium groups, in which the medians were not reached (Fig. 2). The 5-year PFS rates were 73.3% (95% CI, 64.0–84.1), 66.7% (95% CI, 55.5–80.1), and 37.2% (95% CI, 25.2–54.9) and the 5-year OS rates were 78.7% (95% CI, 70.0–88.5), 70.2% (95% CI, 59.3–83.1), and 44.2% (95% CI, 31.6–61.8) (Fig. 2) in the small, medium and large groups, respectively. Compared to those in the small and medium groups, the HRs for PFS in the large group were 3.18 (95% CI, 1.83–5.51) (*P* < 0.001) and 2.43 (95% CI, 1.38–4.28) (*P* < 0.01), and the HRs for OS in the large group were 3.70 (95% CI, 2.03–6.76) (*P* < 0.001) and 2.48 (95% CI, 1.38–4.46) (*P* < 0.05) (Fig. 2).

Among the subgroup cohort of 139 patients treated with R-CHOP regimens, patients in the large group exhibited significantly shorter median PFS (8.8 months; 95% CI, 9.93–NR) and median OS (72.6 months; 95% CI, 18.6–NR) (Fig. 3). Conversely, median PFS and OS were not reached in the other two groups. The 5-year PFS rates were 74.6% (95% CI, 64.6–86.2), 70.0% (95% CI, 57.5–84.2), and 43.3% (95% CI, 28.8–65.2) and the 5-year OS rates were 81.0% (95% CI, 71.8–91.3), 71.4% (95% CI, 66.4–80.6) and 53.3% (95% CI, 38.2–74.5) (Fig. 3) in the small, medium and large groups, respectively. The HRs for PFS in the large group were 2.74 (95% CI, 1.43–5.24) (*P* < 0.01) and 2.34 (95% CI, 1.18–5.22) (*P* < 0.05), and the HRs for OS were 3.21 (95% CI, 1.56–6.57) (*P* < 0.01) and 2.04 (95% CI, 1.01–4.13) (*P* < 0.05) (Fig. 3), respectively, when compared to those in the small and medium groups.

### Univariate and multivariate analyses for PFS and OS

The results of univariate analysis examining the factors influencing PFS and OS in patients with DLBCL are presented in Table 2. Several risk factors were identified for PFS and OS, including older age (≥ 60, PFS: *P* = 0.0003, OS: *P* = 0.0001), high IPI score (≥ 2, PFS: *P* < 0.0001, OS: *P* < 0.0001), elevated LDH levels (> upper limit of normal, PFS: *P* < 0.0001, OS: *P* < 0.0001), high ECOG (≥ 2, PFS: *P* = 0.0003, OS: *P* < 0.0001), advanced clinical stage (Ann Arbor stage Ⅲ–Ⅳ, PFS: *P* < 0.0001, OS: *P* = 0.0003), absence of rituximab treatment (PFS: *P* = 0.008, OS: *P* = 0.009), high neutrophil-to-lymphocyte ratio (NLR) (≥ 4, PFS: *P* < 0.0001, OS: *P* = 0.001), high monocyte count in peripheral blood (≥ 0.6 × 10^9^/L, PFS: *P* = 0.0002, OS: *P* < 0.0001), and enlarged splenic volume (≥ 315 cm^3^, PFS: *P* < 0.0001, OS: *P* < 0.0001).

**Table 2:** Univariate analysis for progression-free survival and overall survival in DLBCL

		PFS			OS	
Risk factors	HR	95% CI	*P-*value	HR	95% CI	*P-*value
Sex (Male)	2.05	0.76–1.94	0.42	1.34	0.81–2.02	0.260
Age (≥ 60)	2.05	1.29–3.25	0.0003	2.65	1.61–4.35	0.0001
Han's classification (non-GCB)	1.39	0.87–2.22	0.173	1.20	0.73–1.98	0.475
IPI (≥ 2)	2.88	1.79–4.62	< 0.0001	3.31	1.97–5.55	< 0.0001
Elevated LDH (> ULN)	3.24	1.97–5.31	< 0.0001	3.64	2.11–6.29	< 0.0001
Ki-67(≥ 90%)	1.00	0.61–1.64	0.992	0.99	0.58–1.68	0.970
ECOG (≥ 2)	2.37	1.49–3.37	0.0003	2.79	1.70–4.56	< 0.0001
Ann Arbor Stage (III-IV)	3.00	1.59–5.02	< 0.0001	2.72	1.57–4.69	0.0003
Extranodal site number (≥ 1)	1.33	0.84–2.13	0.228	1.44	0.87–2.37	0.158
Absence of Rituximab	2.00	1.20–3.03	0.008	2.04	1.20–3.45	0.009
Bone marrow involvement	1.26	0.51–3.13	0.617	1.14	0.42–3.14	0.799
NLR (≥ 4)	2.33	1.47–3.69	< 0.0001	2.99	1.83–4.90	0.001
Monocyte (≥ 0.6 × 10^9^/L)	2.39	1.51–3.80	0.0002	2.43	1.43–4.12	< 0.0001
Splenic volume (≥ 315 cm^3^)	2.82	1.76–4.53	< 0.0001	3.07	1.87–5.06	< 0.0001

*Abbreviations*: *non-GCB* non-germinal center B cell-like, *IPI* International Prognostic Index, *LDH* lactate dehydrogenase, *ULN* upper limit of normal, *ECOG* Eastern Cooperative Oncology Group, *NLR* neutrophil-to-lymphocyte ratio, *DLCBL* diffuse large B-cell lymphoma, *HR* hazard ratio, *CI* confidence interval, *PFS* progression-free survival, *OS* overall survival

Multivariate analysis revealed that older age (≥ 60, HR 1.95; 95% CI, 1.20–3.16; *P* = 0.006), absence of rituximab treatment (HR 1.91; 95% CI, 1.13–3.39; *P* = 0.016), high monocyte count (≥ 0.6 × 10^9^/L, HR 2.06; 95% CI, 1.28–3.34; *P* = 0.003), and enlarged splenic volume (≥ 315 cm^3^, HR 2.05; 95%CI, 1.19–3.53; *P* = 0.009) were independent risk factors for PFS (Table 3). Meanwhile, older age (≥ 60, HR 2.59; 95% CI, 1.53–4.35; *P* < 0.001), absence of rituximab treatment (HR 1.85; 95% CI, 1.05–3.23; *P* = 0.032), high NLR (≥ 4, HR 2.09; 95% CI, 1.22–3.58; *P* = 0.007), high monocyte count (≥ 0.6 × 10^9^/L, HR 2.09; 95% CI, 1.26–3.49; *P* = 0.004), and enlarged splenic volume ((≥ 315 cm^3^, HR 2.19; 95% CI, 1.24–3.86; *P* = 0.007) were independent risk factors for OS in multivariate analysis (Table 3).

**Table 3:** Multivariate analysis for progression-free survival and overall survival in DLBCL

	PFS		OS	
Risk factors	HR (95% CI)	*P-*value	HR (95% CI)	*P-*value
Age (≥ 60)	1.95 (1.20–3.16)	0.006	2.59 (1.53–4.35)	< 0.001
Han's classification (non-GCB)	1.56 (0.96–2.53)	0.075	1.56 (0.91–2.65)	0.103
ECOG (≥ 2)	1.14 (0.66–1.96)	0.638	1.37 (0.77–2.45)	0.289
Ann Arbor Stage (III-IV)	1.52 (0.85–3.35)	0.132	1.37 (0.77–2.45)	0.694
Elevated LDH (> ULN)	1.52 (0.82–2.81)	0.187	1.79 (0.91–3.50)	0.091
Extranodal site number (≥ 1)	1.04 (0.62–1.71)	0.872	1.28 (0.74–2.20)	0.384
Absence of Rituximab	1.96 (1.13–3.39)	0.016	1.85 (1.05–3.23)	0.032
NLR (≥ 4)	1.53 (0.92–2.56)	0.102	2.09 (1.22–3.58)	0.007
Monocyte (≥ 0.6 × 10^9^/L)	2.06 (1.28–3.34)	0.003	2.09 (1.26–3.49)	0.004
Splenic volume (≥ 315 cm^3^)	2.05 (1.19–3.53)	0.009	2.19 (1.24–3.86)	0.007

*Abbreviations*: *non-GCB* non-germinal center B-cell-like, *ECOG* Eastern Cooperative Oncology Group, *LDH* lactate dehydrogenase, *ULN* upper limit of normal, *NLR* neutrophil-to-lymphocyte ratio, *DLCBL* diffuse large B-cell lymphoma, *HR* hazard ratio, *CI* confidence interval, *PFS* progression-free survival, *OS* overall survival

### Peripheral blood inflammatory markers in patients with DLBCL with different splenic volumes

**Fig. 4:**
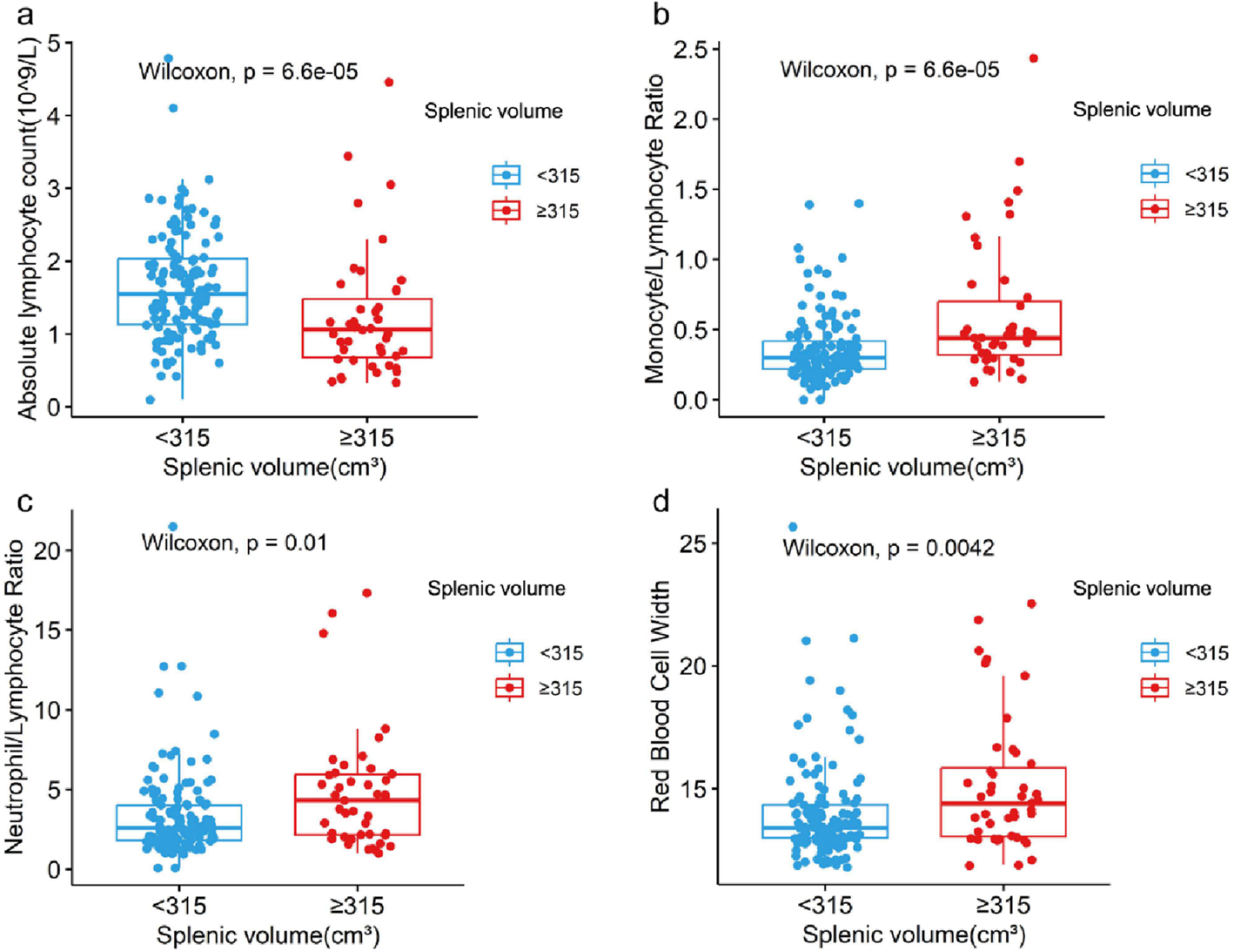
Peripheral blood inflammatory markers in patients with different splenic volume compared to that of those with small splenic volume (< 315 cm^3^). Lymphocytopenia is distinctive in patients with enlarged splenic volume (≥ 315 cm.^3^) (**a**), Monocyte to Lymphocyte ratio (MLR) (**b**), Neutrophil to Lymphocyte ratio (NLR) (**c**), and red blood cell distribution width (RDW) were higher in the enlarged splenic volume group (**d**)

Laboratory data on complete blood counts were obtained from all 175 patients prior to DLBCL treatment. As shown in Fig. 4, lymphocytopenia was significant in patients with enlarged splenic volume (≥ 315 cm^3^) (*P* < 0.001). Monocyte-to-lymphocyte ratio (MLR) and NLR were elevated in the large splenic volume group compared to that in the smaller volume group (≤ 315 cm^3^) (MLR: *P* < 0.001; NLR: *P* < 0.01). Additionally, red blood cell distribution width (RDW) was higher in patients with an enlarged splenic volume (*P* < 0.005).

## Discussion

The spleen, the largest secondary lymphoid organ, plays a significant role in innate and adaptive immunity by housing numerous immune cells including macrophages, dendritic cells (DCs), and subsets of T and B cells [11]. In a study by Guo et al., excessive splenic volume was found to be associated with decreased OS and disease-free survival (DFS) in patients with non-small cell lung cancer (NSCLC) who underwent chemoradiotherapy. Splenic volume was identified as an independent prognostic factor in patients who received this treatment modality [12]. To our knowledge, few studies have examined the association between splenic volume and DLBCL prognosis. In our study, 43 (24.6%) patients demonstrated significant splenic enlargement, of whom 20 (46.5%) had no splenic involvement. Splenic involvement in lymphoma was more prevalent in the large group. Compared to those in the small and medium groups, patients in the large group had shorter PFS and OS when receiving CHOP with or without rituximab. These findings were confirmed using univariate and multivariate analyses.

The mechanisms underlying splenomegaly in patients with DLBCL remain unclear. In healthy individuals, splenic volume is significantly and independently associated with sex, height, and weight [13]. However, in this retrospective study, we found no significant association between the splenic volume and anthropometric parameters. These results suggest that changes in the splenic volume are attributable to the disease itself. Baseline characteristics showed that a higher proportion of patients in the large group had poor performance status, elevated serum LDH levels, advanced disease stage, higher IPI, and a higher probability of bone marrow and spleen involvement. Our data suggest that splenic enlargement is associated with a high tumor volume. Tumor-derived factors such as granulocyte–macrophage colony-stimulating factor, granulocyte colony-stimulating factor, and peptide hormone angiotensin II contribute to the systemic deviation of hematopoiesis [14, 15]. A bias toward immunosuppressive myeloid differentiation characterizes splenic extramedullary hematopoiesis [5]. It is reasonable to expect that more tumor-derived factors are produced in patients with high tumor volumes, thereby promoting splenic extramedullary hematopoiesis and an aberrant anti-cancer immune response driven by increased generation of immunosuppressive cells.

The spleen plays significant roles in various physiological and pathological processes. In the context of cancer, data from various animal models have shown that tumors induce spleen extramedullary hematopoiesis, generating immature myeloid-derived suppressor cells (MDSCs) [16], Ter-119 + CD45-CD71 + erythroblast-like cells (Ter-cells) [17], and CD45 + CD71 + TER119 + erythroid progenitor cells (CD45 + EPCs) [18]. These cells have been found to exert immunosuppressive effects on antitumor immunity by impeding T cell proliferation. Analysis of peripheral blood inflammatory markers revealed that patients with enlarged splenic volumes exhibited distinct lymphocytopenia and elevated MLR, NLR, and RDW. These markers suggest a compromised anti-cancer immune response; however, this interpretation remains speculative without direct functional assays. In clinical practice, a smaller pretreatment splenic volume is associated with a better response to immune checkpoint inhibitors in patients with melanoma [19]. In a mouse cancer model, the abrogation of splenic extramedullary hematopoiesis enhanced the efficacy of anti–PD-L1 therapy [5]. Based on our results, an enlarged splenic volume was associated with poor prognosis.

However, the effects of splenectomy on cancer progression are controversial. For instance, several investigations have indicated that splenectomy does not confer any long-term survival benefits to patients undergoing esophagectomy for esophageal carcinoma [20], gastrectomy for gastric cancer [21], or cytoreductive surgery for advanced/recurrent ovarian cancer [22]. In experimental tumor models, temporary deceleration of tumor growth was observed upon removal of the spleen; however, after 2 weeks, the tumor size in the splenectomy group surpassed that of the sham group, and no survival advantage was discernible in the splenectomy group [16]. The spleen is a crucial immunological organ that plays a significant role in both physiological and pathological processes. In cancer, the spleen dynamically harbors both pro- and anti-tumor immune cells. Splenectomy, which involves removal of the spleen, eliminates pro-cancer immune cells and inevitably destroys anti-cancer immunity. However, other organs, such as the bone marrow, can continually supply additional pro-cancer immune cells, contributing to the complex interplay within the cancer immune system. In tumors, many MDSCs are sequestered in the spleen. After splenectomy, MDSCs are released into the peripheral blood, accumulate, and support angiogenesis within the tumor. These mechanisms may explain the lack of long-term survival benefits associated with splenectomy.

However, our study has some limitations. First, selection bias was unavoidable in this retrospective study. Specifically, we excluded patients who survived but received fewer than four cycles of treatment (e.g., due to patient preference, socioeconomic barriers, or nonfatal toxicity), while retaining those who died or progressed early. This may have enriched the cohort with more unfit individuals and potentially overestimated the true prognostic impact of splenic enlargement, because patients with enlarged splenic volume had a high probability of having ECOG ≥ 2. Additionally, patients lost to follow-up or those with incomplete imaging data were excluded, which could further aggravate the selection bias. Secondly, baseline staging was performed using PET/CT and contrast-enhanced CT scans. In some cases, distinguishing between splenic enlargement and involvement on contrast-enhanced CT can be challenging [23]. In addition, the prognostic value of splenic involvement in patients with DLBCL remains controversial [23–25]. Therefore, large-scale prospective cohort studies are required to validate the prognostic significance of splenic enlargement and its involvement in DLBCL. Third, we could not directly determine whether patients with an enlarged splenic volume had splenic extramedullary hematopoiesis, nor could we provide detailed information on abnormalities in anti-cancer or pro-tumor immunity. Consequently, the direct measurement of biological inflammatory parameters from patient blood samples is required to establish stronger, more definitive, and more reliable associations between splenomegaly and inflammatory imbalances.

## Conclusion

Our study suggests a significant association between enlarged splenic volume (≥ 315 cm^3^) and unfavorable prognosis in patients with DLBCL. Patients with enlarged splenic volumes tended to have shorter PFS and OS. Additionally, the analysis of peripheral blood inflammatory markers indicated that an enlarged splenic volume correlated with significant lymphocytopenia and higher MLR, NLR, and RDW values. These inflammatory markers suggest compromised anti-cancer immune responses and aberrant inflammatory conditions; however, this remains speculative. An enlarged splenic volume might therefore reflect a high tumor burden and impaired anti-cancer immunological reactions; nonetheless, this hypothesis requires direct testing.

## Supplementary Information

Supplementary Material 1.

## Data Availability

The original contributions presented in the study are included in the article; further inquiries can be directed to the corresponding authors.
