# Supplementary material for: Enlarged splenic volume predicts poor survival and is associated with inflammatory imbalance in patients with diffuse large B-cell lymphoma
Source: Blood Res. 2026 May 8;61(1):28. doi: 10.1007/s44313-026-00138-1 (PMC13250030; doi:10.1007/s44313-026-00138-1)
Supplement: Supplementary file 1 — Supplementary Material 1. [file 44313_2026_138_MOESM1_ESM.docx]

SUPPLEMENTARY MATERIALS

for

# Enlarged Splenic Volume Predicts Poor Survival and Inflammatory Imbalance in Patients with Diffuse Large B-Cell Lymphoma

Zongjian Qiu^1,2^, Rifeng Jiang^3^, Shunquan Wu^1,2^, Rong Zhan^1,2^, Xiaomei Hu^4*^**,** Shaoyuan Wang^1,2*^

^1^Fujian Institute of Hematology, Fujian Provincial Key Laboratory on Hematology, Fujian Medical University Union Hospital, Fuzhou, Fujian Province, People’s Republic of China.

^2^ Department of Hematology, Fujian Medical University Union Hospital, Fuzhou, Fujian Province, People’s Republic of China.

^3^Department of Radiology, Fujian Medical University Union Hospital. Fuzhou, Fujian Province, People’s Republic of China.

^4^Department of Pathology, Fujian Medical University Union Hospital, Fuzhou, Fujian Province, People’s Republic of China.

* **Corresponding authors:**

Xiaomei Hu, Department of Pathology, Fujian Medical University Union Hospital, Xinquan Road No.29, Fuzhou, Fujian Province, 30001, People’s Republic of China. Email: [jimemory@163.com](mailto:jimemory@163.com). ORCID: 0000 0003 4276 7026

Shaoyuan Wang, Department of Hematology, Fujian Medical University Union Hospital, Xinquan Road No.29, Fuzhou, Fujian Province, 30001, People’s Republic of China. Email: [shaoyuanwang@fjmu.edu.cn. ORCID](mailto:shaoyuanwang@fjmu.edu.cn. ORCID)*:* 0000-0001-7428-8682

***Other author's email:***

Zongjian Qiu, [lanzhou12@163.com](mailto:lanzhou12@163.com), ORCID: 0009-0007-3298-2714

Shunquan Wu, wsqlx1027@126.com

Rifeng Jiang, 26630706@qq.com

Rong Zhan, 1835659021@qq.com

**Keywords:** prognostic factor; inflammatory markers; imbalanced anti-cancer immunology


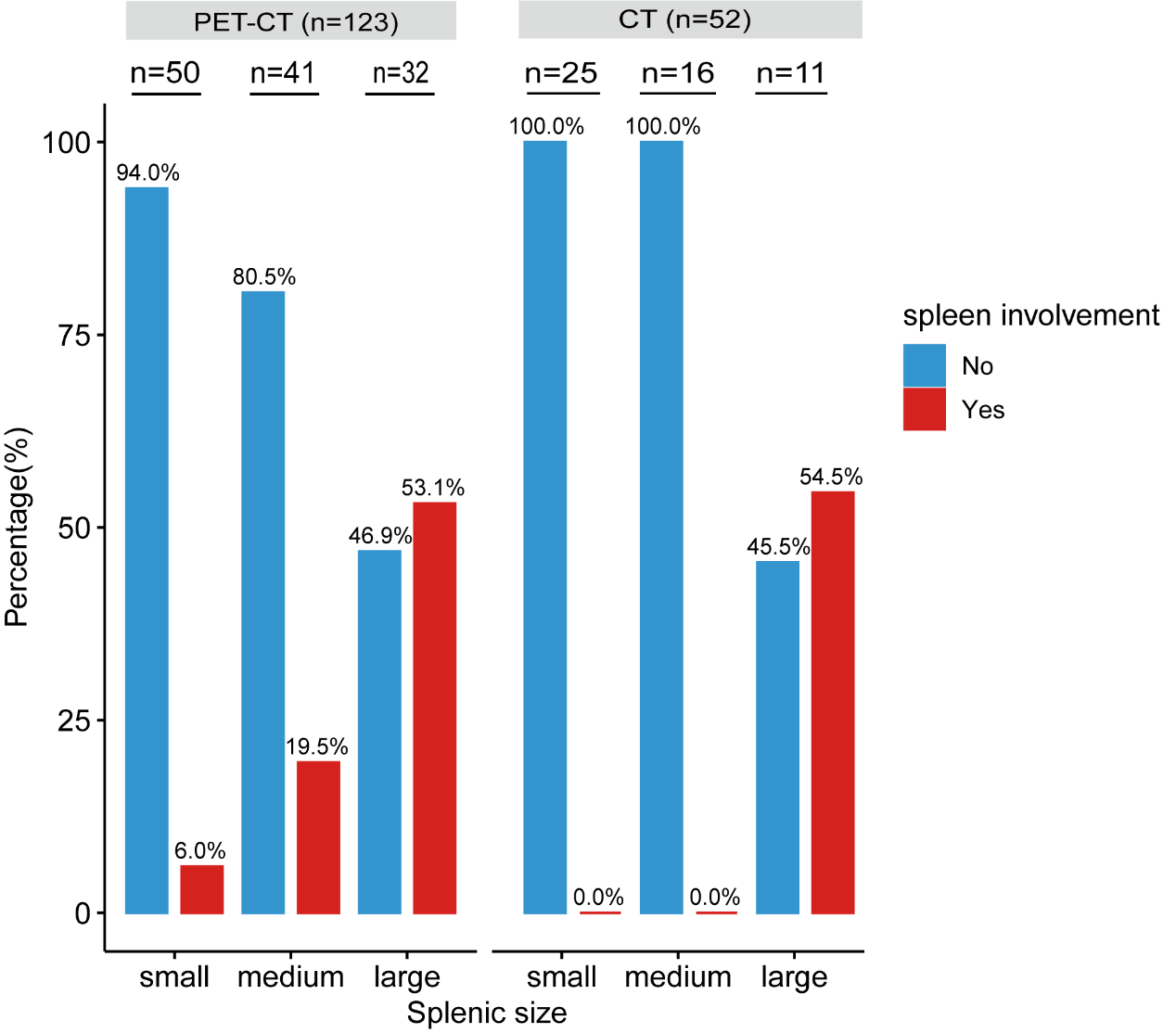


Fig.S1 The status of spleen involvement by DLBCL was confirmed by PET-CT (n=123) or contrast-enhanced CT (n=52) in different splenic size groups


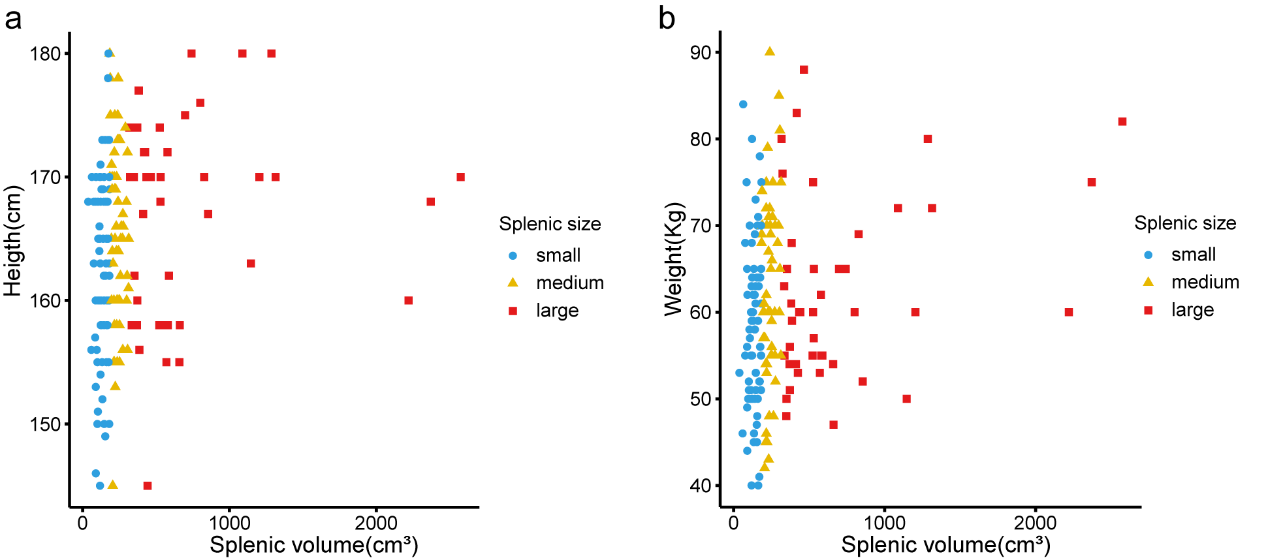


Fig.S2 Spearman's rank correlation analysis the correlation between height and splenic volume (r = 0.051, P = 0.503) (a), as well as between weight and splenic volume (r = 0.089, P = 0.240) (b)
